# A Rare Concurrence of Leiomyomatosis Peritonealis Disseminata, Leiomyosarcoma of the Pelvis and Leiomyomatous Nodule of the Liver

**DOI:** 10.1155/2016/3025432

**Published:** 2016-02-22

**Authors:** Aung Myint Tun, Nay Min Tun, Kyaw Zin Thein, Ei Ei Naing, Shah Giashuddin, Maxim Shulimovich

**Affiliations:** ^1^Department of Medicine, The Brooklyn Hospital Center, Brooklyn, NY 11201, USA; ^2^Health Pavilion North Cancer Center, Fayetteville, NC 28311, USA; ^3^Department of Oncologic Emergency Medicine, The University of Texas MD Anderson Cancer Center, Houston, TX 77030, USA; ^4^St. George's University School of Medicine, St. George's, Grenada; ^5^Department of Pathology and Laboratory Medicine, The Brooklyn Hospital Center, Brooklyn, NY 11201, USA; ^6^Department of Medicine, Division of Hematology and Oncology, The Brooklyn Hospital Center, Brooklyn, NY 11201, USA

## Abstract

Leiomyomatosis peritonealis disseminata (LPD) is a rare entity that is characterized by the presence of multiple subperitoneal or peritoneal smooth muscle nodules throughout the peritoneal surface mimicking a malignant process. LPD follows a benign course in general, and it is often found incidentally during abdominal surgery. There have been reported cases of LPD with malignant degeneration although the association is uncertain. Concurrent finding of LPD and leiomyosarcoma of the pelvis is very rare that could be coincidental, malignant transformation of LPD to leiomyosarcoma, or progression of undetected primary leiomyosarcoma. There are only a few previously reported cases in the literature. Herein, we report a case of 56-year-old woman with a history of leiomyoma of uterus who presented with progressive abdominal swelling secondary to mass lesions in the pelvis. The patient underwent exploratory laparotomy and debulking of the tumors, and the histologic examination of the tumors revealed coexistence of LPD and leiomyosarcoma. After recovery from the operation, core needle biopsy of the superficial, residual liver mass was obtained to investigate potential liver metastasis, and the histopathologic findings are consistent with leiomyoma which represents the first simultaneous occurrence of LPD, leiomyosarcoma, and leiomyomatous nodule of the liver.

## 1. Introduction

Leiomyomatosis peritonealis disseminata (LPD) is an unusual condition and benign in nature; however, very rarely, LPD may degenerate into malignancy [1–3]. Although there have been a few reported cases of LPD associated with leiomyosarcoma in the literature previously, simultaneous detection of LPD, leiomyosarcoma, and leiomyomatous nodule of the liver has never been reported, which represents the first reported case.

## 2. Case Description

**Figure 1:**
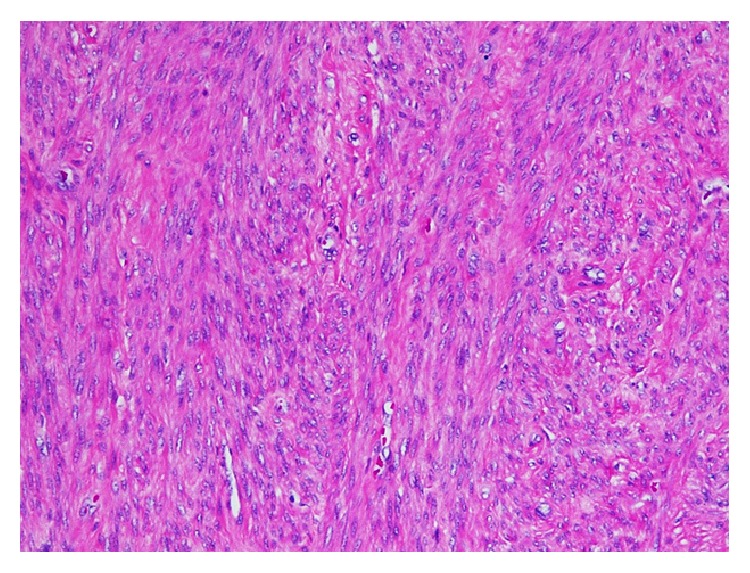
Section from hysterectomy specimen shows proliferation of spindle cells with uniform nuclei (Hematoxylin and Eosin stain, 100x magnification).

A 56-year-old African American woman was referred to the hospital for elective excision of a pelvic mass. The patient initially presented with increasing abdominal distension and bloating, especially after eating food. Imaging studies done at an outside facility revealed a pelvic mass, and she was sent to the hospital for removal of the mass. The patient had a past surgical history of total abdominal hysterectomy in 2005, and the histologic examination revealed leiomyoma of the uterus (Figure 1). An exploratory laparotomy was performed, and it revealed multiple pelvic masses, some of which were greater than 10 cm, frozen pelvis with ascites measuring 2 liters, and extensive abdominoperitoneal carcinomatosis many of which were smooth-surfaced suggestive of benign myomatous lesions. There was also a mass lesion on the surface of the liver which could not be removed or biopsied due to a risk of bleeding. A nearly complete cytoreductive surgery was performed, leaving a 1 cm residual mass in the pelvis and a 2 cm lesion in the liver. The patient was discharged on the seventh postoperative day without any adverse event.

**Figure 2:**
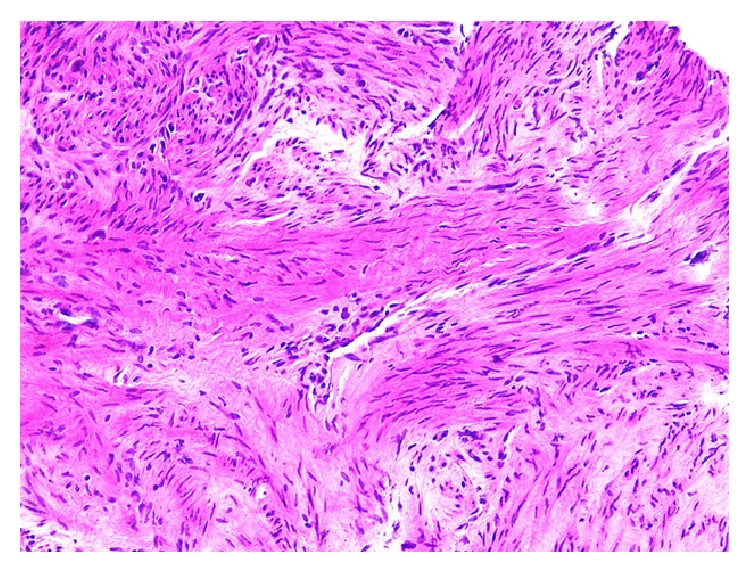
Section from smaller pelvic masses shows proliferation of uniform spindle shaped cells without significant atypia, mitosis, or necrosis (Hematoxylin and Eosin stain, 40x magnification).

**Figure 3:**
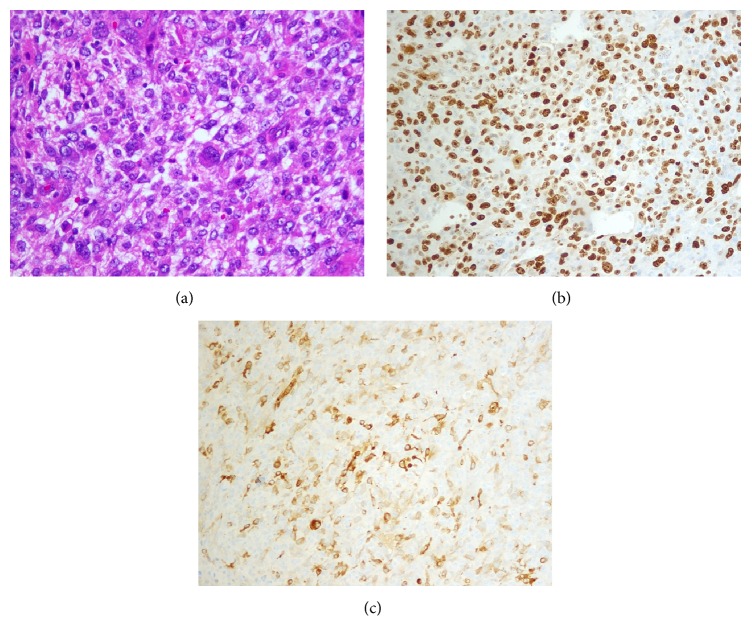
(a) Section from pelvic mass shows pleomorphic cells (Hematoxylin and Eosin stain, 100x magnification). (b) Ki-67 immunostaining showing high proliferative index (more than 50%) (100x magnification). (c) Immunostaining for Smooth Muscle Actin (SMA) showing immunoreactive pleomorphic cells (Smooth Muscle Actin stain, 100x magnification).

**Figure 4:**
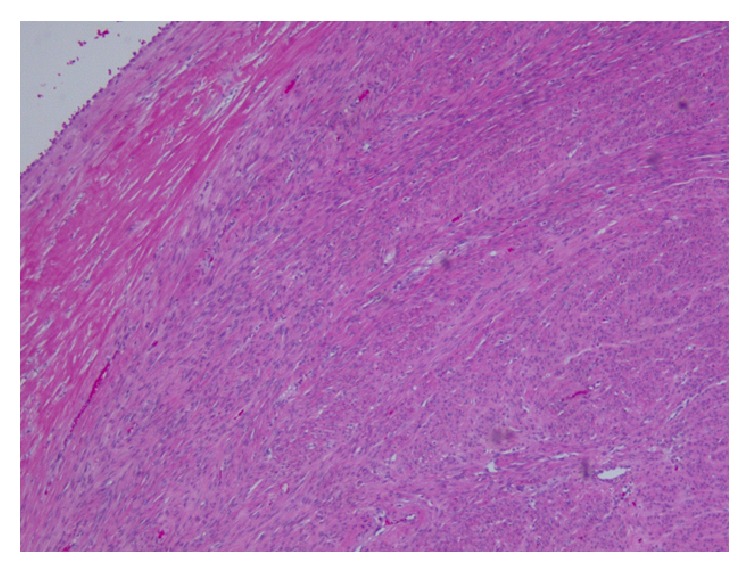
Section from one of peritoneal nodules showing proliferation of spindle cells without atypia, mitosis, or necrosis (Hematoxylin and Eosin stain, 100x Magnification).

**Figure 5:**
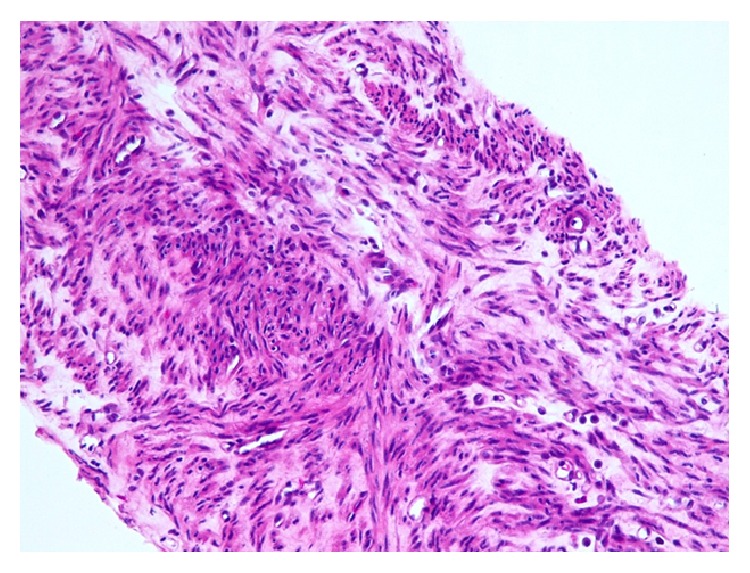
Core biopsy of the liver mass showing proliferation of bland spindle cells without mitosis or necrosis (Hematoxylin and Eosin stain, 100x magnification).

**Figure 6:**
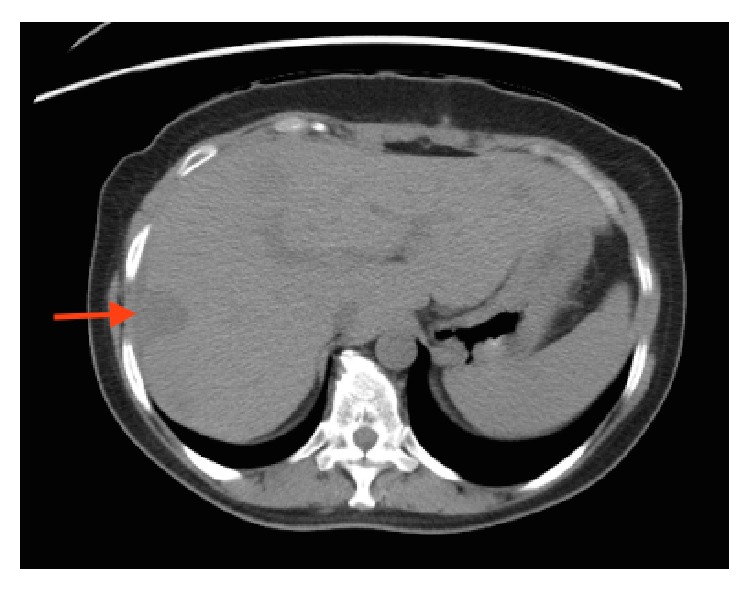
Axial section of CT scan showing the nodule in the liver which represents benign leiomyomatous nodule (red arrow).

Intraoperative frozen section of mass lesions revealed spindle cell tumor. The final pathologic examination of the smaller 5.2 cm × 4.0 cm × 3.0 cm abdominal pelvis mass was reported as leiomyoma (Figure 2) and the larger 14 cm × 10 cm × 10 cm abdominal mass revealed pleomorphic cells with >20 mitotic figures per high power fields (Figure 3(a)) which is consistent with high-grade leiomyosarcoma. Ki-67 is 70% (Figure 3(b)); tumor cells were positive for smooth muscle actin (Figure 3(c)), myosin heavy chain, and vimentin but negative for AE1/AE3, CD117, S100, CD99, ER, calretinin, desmin, estrogen and progesterone receptor, and CD34. The histologic examination of nodules of the peritoneum, omentum, and smaller pelvic masses revealed leiomyomatous deposits (Figure 4) consistent with LPD. Because of recurrence of abdominal distension, CT scan of the chest, abdomen, and pelvis was obtained one month after the operation which revealed a 11 cm × 5.6 cm × 8 cm pelvic mass with peritoneal seeding, lung, and liver nodules. CT guided core biopsy of the residual 2 cm mass located at posterior right dome of the liver (Figure 6) was performed to evaluate for potential liver metastasis, and it was unexpectedly found to be leiomyoma without any features of leiomyosarcoma. The liver biopsy was also reviewed by the pathologist from another institution who concurred with a diagnosis of leiomyoma (Figure 5).

The patient was treated for metastatic leiomyosarcoma because of radiologic findings which showed metastatic nodules in the lungs and the liver. She was started on gemcitabine and docetaxel along with hematopoietic growth factor support.

Although the patient was managed with chemotherapy and supportive care, she expired approximately 5 months after the diagnosis due to rapid progression of the malignancy.

## 3. Discussion

LPD was first described in 1952 by Willson and Peale [3]. It is a rare disorder that occurs primarily in premenopausal women. It is often associated with pregnancy and use of contraceptive pills [4]. Actual incidence of LPD could be underestimated due to its asymptomatic nature, and majority of the cases are incidentally found from abdominal surgeries and imaging procedures.

The etiology and pathogenesis of LPD remain uncertain. Exposure to estrogen is postulated as prior reported cases were related to pregnancy and use of oral contraceptive pills, and most of them resolved after withdrawal of hormonal exposure. On the contrary, there are also reported cases that are not associated with pregnancy, oral contraceptive pill use, or any other estrogen exposure, which may perhaps be from increased sensitivity of LPD nodules to estrogen. Increased expression of estrogen and progesterone receptor in LPD nodules could further support hormonal hypothesis [4]. It is generally accepted that estrogen induced metaplasia of pluripotent mesenchymal stem cells into leiomyocytes plays an important role in the pathogenesis of LPD. However, failure to identify stem cells in LPD nodules challenges the hypothesis of metaplasia [4]. Metastatic peritoneal seeding is another hypothesis, but subperitoneal localization of the nodules makes it less likely [4]. Furthermore, there have been reported cases that are related to endometriosis and prior morcellation of leiomyoma, but more studies are yet to prove it [5, 6].

LPD is characterized by benign, multiple, small, white-gray subperitoneal nodules varying in size from a few millimeters to several centimeters on or beneath the peritoneal surface of the uterus, fallopian tubes, ovaries, small and large intestines, cul-de-sac, and mesentery and in the retroperitoneum [7–9]. Microscopically, LPD is characterized by round nodules composed of mature fusiform smooth muscle cells that are arranged in interdigitating fascicles [4]. The nodules lack cell atypia, nuclear polymorphism, hyperchromasia, and tumor cell necrosis with mitotic figures of less than 3/10 high power field [4].

In contrast, benign metastasizing leiomyoma (BML) usually manifests as one or more pulmonary nodules, and when it arises in the pelvis and abdomen, it generally presents as one or two larger nodules near the region of round ligament or iliac veins [10]. In order to diagnose BML, all smooth muscle tumors must be thoroughly examined histologically and judged as morphologically benign [10]. In addition, it is important to exclude metastatic low grade leiomyosarcoma by meticulous sampling of the pathology specimen before diagnosis of BML is made [11]. Nucci et al. published the associated, specific clonal aberrations in BML, especially 19q and 22q terminal deletions, which may be used as a tool to confirm BML in the future [12]. In this case, labelling as BML for the hypodense lesion in the liver is debatable. Even though thorough microscopic examination of liver nodule revealed leiomyoma, the sample was obtained by core needle biopsy which may not represent the actual pathology of the liver nodule. The presence of leiomyosarcoma further challenges the diagnosis of BML of the liver.

Most LPD are benign in nature; however, it can rarely be associated with leiomyosarcoma [4]. It is postulated that LPD could degenerate into malignancy [1–3], yet more studies, which perhaps include cytogenetic and molecular features, are needed to prove the hypothesis. Although there have been previous reported cases of LPD associated with leiomyosarcoma in the literature, simultaneous detection of LPD, leiomyosarcoma, and metastatic hepatic leiomyoma has never been reported in the literature. Simultaneous detection of LPD and leiomyosarcoma, as seen in our case, could represent coincidental finding, malignant transformation of LPD to leiomyosarcoma, or progression of undetected primary leiomyosarcoma [4].

Most cases of LPD can be managed with conservative approach, without extensive surgery, as spontaneous regression has often been described in LPD, especially after withdrawal of hormonal stimulus (surgical castration, postpartum, stopping contraceptive pills) [4]. On the other hand, more aggressive approach (extensive surgery and closer monitoring) is recommended for LPD with high risk of malignant degeneration such as no exposure to exogenous or increased endogenous estrogen, no history of uterine leiomyomas, and negative estrogen and progesterone expression in the LPD nodules [4].

## 4. Conclusion

LPD is a rare disease and mostly has asymptomatic nature. Concurrence of LPD, leiomyosarcoma, and metastatic leiomyoma of the liver has never been reported previously. Moreover, it is exceptionally rare to have LPD and leiomyosarcoma simultaneously. It is important to note that malignant degeneration of LPD is a hypothesis, and studies are yet to prove it.

## References

[1] Raspagliesi F., Quattrone P., Grosso G., Cobellis L., Di Re E. (1996). Malignant degeneration in leiomyomatosis peritonealis disseminata. *Gynecologic Oncology*.

[2] Sharma P., Chaturvedi K. U., Gupta R., Nigam S. (2004). Leiomyomatosis peritonealis disseminata with malignant change in a post-menopausal woman. *Gynecologic Oncology*.

[3] Willson J. R., Peale A. R. (1952). Multiple peritoneal leiomyomas associated with a granulosa-cell tumor of the ovary. *American Journal of Obstetrics & Gynecology*.

[4] Bekkers R. L. M., Willemsen W. N. P., Schijf C. P. T., Massuger L. F. A. G., Bulten J., Merkus J. M. W. M. (1999). Leiomyomatosis peritonealis disseminata: does malignant transformation occur? A literature review. *Gynecologic Oncology*.

[5] Yoshida A., Nii S., Matsushita H., Morii Y., Watanabe K., Wakatsuki A. (2015). Parasitic myoma in women after laparoscopic myomectomy: a late sequela of morcellation?. *Journal of Obstetrics and Gynaecology*.

[6] Kuo T., London S. N., Dinh T. V. (1980). Endometriosis occurring in leiomyomatosis peritonealis disseminata. Ultrastructural study and histogenetic consideration. *American Journal of Surgical Pathology*.

[7] Gompel C., Silverberg S. G. (1994). *Pathology in Gynaecology and Obstetrics*.

[8] Enzinger F. M., Weiss S. W. (1988). *Soft Tissue Tumors*.

[9] Kurman R. J. (1994). *Blaustein's Pathology of the Female Genital Tract*.

[10] Carter D., Eggleston J. C., Mills S. E. (1999). *Diagnostic Surgical Pathology*.

[11] Awonuga A. O., Shavell V. I., Imudia A. N., Rotas M., Diamond M. P., Puscheck E. E. (2010). Pathogenesis of benign metastasizing leiomyoma: a review. *Obstetrical and Gynecological Survey*.

[12] Nucci M. R., Drapkin R., Cin P. D., Fletcher C. D. M., Fletcher J. A. (2007). Distinctive cytogenetic profile in benign metastasizing leiomyoma: pathogenetic implications. *The American Journal of Surgical Pathology*.

